# lncRNA NORAD promotes lung cancer progression by competitively binding to miR-28-3p with E2F2

**DOI:** 10.1515/med-2022-0538

**Published:** 2022-09-28

**Authors:** Wenjun Mao, Shengfei Wang, Ruo Chen, Yijun He, Rongguo Lu, Mingfeng Zheng

**Affiliations:** Department of Cardiothoracic Surgery, The Affiliated Wuxi People’s Hospital of Nanjing Medical University, Wuxi, 214023, Jiangsu, China; Department of Cardiothoracic Surgery, The Affiliated Wuxi People’s Hospital of Nanjing Medical University, No. 299 Qingyang Road, Wuxi, 214023, Jiangsu, China

**Keywords:** lung cancer, lncRNA NORAD, miR-28-3p, E2F2, competitive endogenous RNA cell proliferation

## Abstract

Lung cancer (LC) is a prevailing primary tumor in the lung. lncRNA non-coding RNA activated by DNA damage (NORAD) is a popular target in human cancers. This experiment is designed to probe the mechanism of lncRNA in LC progression. NORAD expression in normal lung epithelial cells and LC cells was examined and then silenced to assess its effect on LC cell proliferation, invasion, and migration. Subcellular localization of NORAD was analyzed through online databases and then corroborated by fractionation of nuclear and cytoplasmic RNA assay. The target binding relations between NORAD and miR-28-3p and between miR-28-3p and E2F2 were verified. Eventually, LC cells with NORAD silencing were transfected with miR-28-3p inhibitor or pcDNA3.1-E2F2 to measure LC cell proliferation, invasion, and migration. NORAD was overexpressed in LC cells and NORAD knockout led to suppressed LC cell proliferation, invasion, and migration. Besides, NORAD targeted miR-28-3p and miR-28-3p targeted E2F2 transcription. Inhibiting miR-28-3p or overexpressing E2F2 could both annul the inhibitory role of si-NORAD in LC cell proliferation, invasion, and migration. Generally, our findings demonstrated that NORAD competitively bound to miR-28-3p with E2F2, to promote LC cell progression.

## 1 Introduction

Lung cancer (LC) is a malignant neoplasm with surprisingly high incidence and mortality across the world [1]. Although LC in the early phase is hopefully curable, most cases of LC are diagnosed in the late phase, which is accompanied by unavailing treatment and a disappointing prognosis [2]. LC is closely associated with cigarette smoking, genetic factors, air pollution, secondhand smoking, chronic infection, and occupational exposure [3]. LC has a wide diffusion and its management is not always simple [4]. The late diagnosis and insufficient therapeutic options are major reasons for demanding elaborate knowledge of LC [5]. Therefore, exploring novel biomarkers could benefit LC treatment as it helps to discover new medicines, therapies, and technologies [6].

Long non-coding RNAs (lncRNAs) are a group of non-protein-coding transcripts that function as pro-oncogenes or tumor suppressors in LC cells during the procedures of transcription, post-transcription, and epigenetics by controlling molecular or genetic expression [7]. lncRNA non-coding RNA activated by DNA damage (NORAD) is aberrantly expressed and acts as an oncogene in a wide range of human carcinomas by manipulating cancer cell survival, development, metastasis, and expansion [8]. For instance, NORAD is abundantly expressed in colorectal cancer, osteosarcoma, and prostate cancer and is responsible for cancer cell viability, mobility, invasiveness, and dissemination, as well as unwelcome clinic consequences [9,10,11]. NORAD is associated with lung pathology [12]. Besides, NORAD is activated in non-small cell lung cancer (NSCLC) and predicts poor overall survival and lymph node metastasis in an advanced stage of NSCLC [13]. Interestingly, NORAD could exacerbate pancreatic cancer malignancy via the competitive endogenous RNA (ceRNA) network involving the downstream microRNA (miR) and mRNA [14]. Research on genes and miRNA is of high importance in the area of precision medicine [15,16]. MiRs are necessarily correlated to cancer amelioration or malignancy by altering gene expression, modulating cancer cellular biological behaviors and molecular pathways, sustaining homeostasis, and regulating drug or chemotherapy resistance [17]. The miR-28 cluster, such as miR-28-5p, is noted to negatively regulate NSCLC progression [18]. miR-28-3p is a widely accepted anti-tumor gene as it is downregulated in many malignancies, including nasopharyngeal carcinoma and colorectal cancer, and is predictive of reduced cancer cell mobility and invasiveness [19,20]. However, the role of miR-28-3p in LC is poorly understood. On the other hand, miR-28-3p works like a sponge of its upstream LINC02381 in the ceRNA interaction to promote cervical adenocarcinoma development [21]. Inhibition of E2F transcription factor 2 (E2F2) is conducive to LC amelioration [22]. E2F2 could function as the downstream mRNA to mediate hepatocellular carcinoma [23]. The aforementioned evidence strengthened our faith and confidence to hypothesize that the NORAD/miR-28-3p/E2F2 ceRNA network was involved in LC development.

## 2 Materials and methods

### 2.1 Cell culture and treatment

Human LC cell lines (A549, Calu-3, NCI-H1299, and H1650) and human normal lung epithelial cell line (BEAS-2B) (all from American Type Culture Collection, Manassas, VA, USA) were incubated in Dulbecco’s modified Eagle’s medium/Ham’s Nutrient Mixture F12 (DMEM/F12, Gibco, Life Technologies, Carlsbad, CA, USA) with 10% (v/v) fetal bovine serum (FBS) and 1% (v/v) penicillin streptomycin-glutamine (100×, both Gibco, Life Technologies) at 37°C with 5% CO_2_.

### 2.2 Cell transfection

miR-28-3p-inhibitor, small interfere (si) RNA targeting NORAD (si-NORAD), plasmid overexpressing E2F2 (pcDNA3.1-E2F2), and the corresponding controls (miR-28-3p-negative control (NC), si-NC, and pcDNA3.1-NC) (all from RiboBio Co., Ltd, Guangzhou, Guangdong China) were transfected into cells by Lipofectamine 2000 (Invitrogen Inc., Carlsbad, CA, USA) following the manufacturers’ instructions. After 48 h of transfection, all cells were preserved for further analysis.

### 2.3 Reverse transcription quantitative polymerase chain reaction (RT-qPCR)

The total RNA was extracted from cells using the TRIzol reagent (T9108, Takara, Toyko, Japan) complying with its instructions. The miRNA First Strand cDNA Synthesis (Tailing Reaction) kits (B532451-0020, Sangon Biotech Co., Ltd, Shanghai, China) were employed to assess the miR expression and reverse transcribe the total RNA into the cDNA. The lnRrcute lncRNA cDNA Chain synthesis kits (KR202, TianGen Biotech Co., Ltd, Beijing, China) were appointed for reverse transcription of lncRNA and genes. Next RT-qPCR reactions were carried out using the SYBR^®^ Premix Ex Taq^TM^ II (Perfect Real Time) kits (DRR081, Takara, Toyko, Japan). A total of 20 µL of system (4 µL of template cDNA, 0.8 µL of forward primers [10 µmol/L], 0.8 µL of reverse primers [10 µmol/L], 10 µL of SYBR^®^ Premix Ex TaqTM II, and 4.4 µL of sterile water) was supplemented into each well of 96-well plates. Each sample was committed three times. The processes of amplification response included pre-denaturation at 95°C for 30 s, and 39 cycles of denaturation at 95°C for 5 s, annealing at 60°C for 18 s, and extension at 72°C for 15 s. The fluorescence signal of the dissolution curve was collected. The experimental data were analyzed using the relative quantification method. Ct value of each well was recorded, with glyceraldehyde-3-phosphate dehydrogenase (GAPDH) or U6 as the internal reference, and the relative expression of genes was calculated using the 2^−ΔΔCt^ method. ΔΔCt = (ΔCt Experimental group target genes – ΔCt Experimental group housekeeper genes) – (ΔCt Control group target genes – ΔCt control group housekeeper genes). The primers used are shown in Table 1.

**Table 1:** Primer sequence of RT-qPCR

Name of primer	Forward Primer (5′−3′)	Reverse Primer (5′−3′)
LncRNA-NORAD	AGTTCCGGTCCGGCAGAGAT	GCCCCTCTGC TGCCAACCTA
miR-28-3p	CGCGCACTAGATTGTGAGCT	AGTGCAGGGTCCGAGGTATT
E2F2	AAAGGGTCTTATGCACTGGA	CAAGTTTGCTTCTCTGAGCC
U6	CTCGCTTCGGCAGCACA	AACGCTTCACGA ATTTGCGT
GAPDH	TGCACCACCAACTGCTTAGC	GGCATGCACTGTGGTCATGAG

Note: RT-qPCR, reverse transcription-quantitative polymerase chain reaction; LncRNA, long non-coding RNA; NORAD, non-coding RNA activated by DNA damage; miR, microRNA; E2F2, E2F transcription factor 2; GAPDH, glyceraldehyde-3-phosphate dehydrogenase.

### 2.4 Western blot analysis

Tissues or cells with different treatments were lysed for 30 min using lysis buffer (Beijing Biolab Technology Co., Ltd, Beijing, China, lysate:phenylmethanesulfonyl fluoride = 50:1, with phosphatase inhibitor added for detection of phosphorylated proteins, lysate:phosphatase inhibitor = 100:1). After centrifugation, the supernatant was isolated and placed into a 0.5 mL centrifuge tube. The content and concentration of each sample were determined using the bicinchoninic acid kits (Boster Biological Technology Co., Ltd, Wuhan, Hubei, China), and the samples were loaded onto sodium dodecyl sulfate-polyacrylamide gel electrophoresis and then transferred onto polyvinylidene fluoride membranes. Membranes were blocked with 1% bovine serum albumin for 2 h and incubated with primary antibodies: E2F2 (ab138515, 1:1,000, Abcam Inc., Cambridge, MA, USA) and GAPDH (ab181602, 1:1,000, Abcam) rabbit monoclonal antibodies at 4°C overnight. After 24 h, the membranes were rinsed, followed by incubation with horseradish peroxidase-labeled goat anti-rabbit secondary antibody (ab205718, 1:1,000, Abcam) for 1 h. Next protein samples were removed from the membranes and visualized via incubation with an enhanced chemiluminescence assay reagent (EMD Millipore, Billerica, MA, USA). The bands from each group in the western blot images were quantified for grey value using Image-Pro Plus 6.0 (Media Cybernetics, San Diego, CA, USA), with GAPDH as the internal reference. Each procedure was repeated three times.

### 2.5 Cell counting kit-8 (CCK-8) method

Cell proliferation in different groups was evaluated by the CCK-8 method. Cells (1 × 10^4^ cells/well) were seeded into 96-well plates in the light of the instructions of CCK-8 kits (Beyotime Biotechnology Co., Ltd, Shanghai, China) with 10 μL of CCK-8 solution supplemented into each well at 37°C with 5% CO_2_ and 95% humidity. Cell proliferation was measured using the CCK-8 method as the optical density at the wavelength of 450 nm was detected using a microplate reader (Bio-Rad 680, Hercules, CA, USA) during the exposure.

### 2.6 Transwell assays

Transwell assay was conducted to examine cell invasion and migration in different groups. Briefly, 5,000 cells resuspended in the serum-free medium were added into the Transwell incubator and placed in the cell medium supplemented with 5% FBS for overnight cultivation. For cell invasion experiments, the polycarbonate membrane between the apical chamber and the basolateral chamber was coated with Matrigel (Shanghai Yanhui Biotechnology Co., Ltd, Shanghai, China) to mimic the extracellular matrix *in vivo*. If the cells want to enter the basolateral chamber, they need to secrete matrix metalloproteinases to degrade the Matrigel before they can pass through the polycarbonate membrane. No Matrigel was used for the migration assay. The Transwell chamber was placed in a 24-well plate that contained 500 μL of DMEM containing 10% FBS. After 24 h, the cells were secured with 4% paraformaldehyde, stained using GIMSA solution, and observed under a microscope (Olympus, Tokyo, Japan) for the counting of 3 random observation points.

### 2.7 5-Ethynyl-2′-deoxyuridine (EDU) assay

Cells (1 × 10^5^ cells/well) at the logarithmic growth stage were seeded in 96-well plates. The appropriate amount (50 mM) of EdU medium (RiboBio, Guangzhou, China) was prepared by diluting EdU solution in a cell medium at a ratio of 1,000:1. Next 100 µL EdU dilution solution was supplemented to each well using a pipette and then cultivated for 2 h. Afterward, the medium was discarded, and the cells were washed continuously with PBS 1–2 times (5 min/each time). Cells in each well were cultured with 50 µL of cell fixation solution (PBS consisting of 4% paraformaldehyde) for 30 min with the medium removed. Cells were secured by 4% paraformaldehyde, stained by 4′,6-diamidino-2-phenylindole for 5 min, and photographed by a microscope (Olympus) to record cell proliferation. Finally, 6–10 randomly selected visual fields were observed and counted under a fluorescent microscope. EdU labeling rate (%) = Number of positive cells/(Number of positive cells + Number of negative cells) × 100%. Eventually, the data were statistically analyzed.

### 2.8 Fractionation of nuclear and cytoplasmic RNA assay

A549 cells at logarithmic growth phase preserved in a –80°C refrigerator were removed onto the ice for 5 min, added with 1 mL of hypotonic lysis buffer (M334-100ML, LANSO Biotechnology Co., Ltd, Jiaxing, Zhejiang, China, 10 mM Hepes-NaOH, pH 7.9, 10 mM KCl, 1.5 mM MgCl_2_, 0.2 Nonidet P-40 [NP-40], proteinase inhibitor), blown up, percussed at 4°C for 20 min and centrifuged at 16,000×*g* at 4°C for 5 min. And then cytoplasm was collected and washed 3 times with hypotonic solution, added with 0.5 mL of high salt lysis buffer (Shanghai Xinyu Biotech Co., Ltd, Shanghai, China, 10 mM tris-HCl, pH 7.6, 420 mM NaCl, 0.5% NP-40, and 1 mM dithiothreitol, 2 mM MgCl_2_ plus protease inhibitors), blown up, removed onto the ice for 20 min and centrifuged at 16,000×*g* at 4°C for 5 min. Then, the nucleoplasm was collected, washed once with high salt solution, and cultivated with 0.5 mL of 0.25 M HCl at 4°C overnight with percussion. Next the nucleoplasm was centrifuged at 15,000×*g* for 20 min, and the obtained supernatant was the nucleus.

### 2.9 Dual-luciferase reporter gene assay

The binding sites of NORAD and miR-28-3p and the binding sites of miR-28-3p and E2F2 were predicted via the StarBase database (http://starbase.sysu.edu.cn/index.php). The synthesized sequences of wild type (WT) and mutant type (MUT) of NORAD and E2F2 gene fragments containing miR-28-3p binding sites were inserted into pmirGLO reporter plasmids (Beijing Huayueyang Biotechnology, Beijing, China). Then, the constructed luciferase reporter plasmids were co-transfected with mimic NC or miR-28-3p mimic into HEK293 cells (Shanghai Beinuo Biotechnology, Shanghai, China) for 48 h. Then, the cells were harvested and lysed. Luciferase activity was determined using the dual-luciferase assay kits (K801-200; Biovision, Mountain View, CA, USA).

### 2.10 Statistical analysis

SPSS 19.0 software (IBM Corp. Armonk, NY, USA) was appointed for data analysis, and GraphPad Prism 8.0 software (GraphPad Software Inc., San Diego, CA, USA) was appointed for graphing. Measurement data were presented as mean value ± standard deviation. All data were inspected with normality distribution and homogeneity test of variance. One-way or two-way analysis of variance (ANOVA) was appointed for comparison analysis among multiple groups, and Tukey’s multiple comparisons test was used for post-test of data. The *p* value was attained using a two-tailed test and a value of *p* < 0.05 indicated a significant difference and *p* < 0.01 indicated a greatly significant difference. Data results were taken at least three times in parallel for each group to ensure statistical significance.

## 3 Results

### 3.1 NORAD is strongly expressed in LC cell lines and NORAD knockdown in LC cells inhibits cancer cell proliferation, invasion, and migration

**Figure 1:**
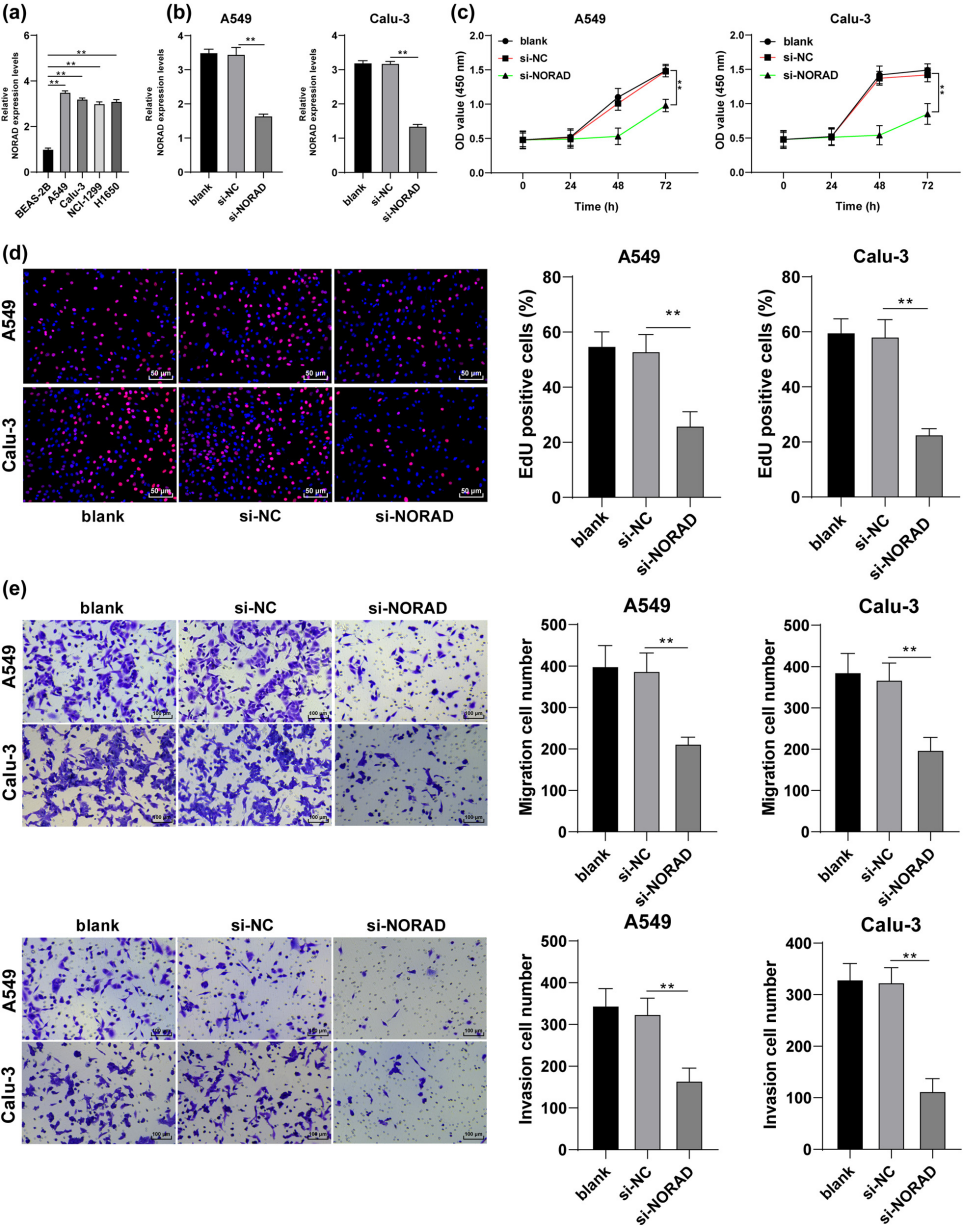
NORAD is strongly expressed in LC cell lines and NORAD knockdown in LC cells inhibits cancer cell proliferation, invasion, and migration. (a) NORAD expression in human normal lung epithelial cells (BEAS-2B) and LC cell lines (A549, Calu-3, NCI-H1299, and H1650) was assessed by RT-qPCR. si-NC or si-NORAD was transfected into A549 and Calu-3 cells. (b) NORAD expression in A549 and Calu-3 cells was assessed by RT-qPCR. (c and d) LC cell proliferation was tested by the CCK-8 method (c) and EdU assay (d). (e) LC cell invasion and migration were detected by Transwell assays. The independent cell experiments were repeated three times. The results were presented as mean value ± standard deviation. Two-way ANOVA was appointed to analyze the data in panel c and one-way ANOVA was used to analyze the data in panels a, b, and d–e. Tukey’s multiple comparisons test was applied for the post hoc test. ** *p* < 0.01.

According to the relevant literature, NORAD is robustly expressed in hepatocellular carcinoma cells [24]. RT-qPCR was employed to evaluate NORAD expression in BEAS-2B cells and the LC cell lines (A549, Calu-3, NCI-H1299, and H1650). And the results revealed that NORAD was overexpressed in LC cells (*p* < 0.05, Figure 1a), with the highest expression in A549 and Calu-3 cells. As a result, A549 and Calu-3 cells were selected for the subsequent experiments. si-NORAD was injected into A549 and Calu-3 cells to reduce NORAD expression in LC cells (*p* < 0.05, Figure 1b). CCK-8 method and EdU assay were appointed to test cell proliferation, and our findings discovered that the proliferation rate of LC cells in the si-NOARD group was reduced compared to that in the si-NC group (*p* < 0.05, Figure 1c and d). Transwell assays were appointed to determine LC cell invasion and migration, which unveiled that compared with the si-NC group, the si-NORAD group had declined cell invasion and migration (*p* < 0.05, Figure 1e).

### 3.2 NORAD targets miR-28-3p in LC cells

**Figure 2:**
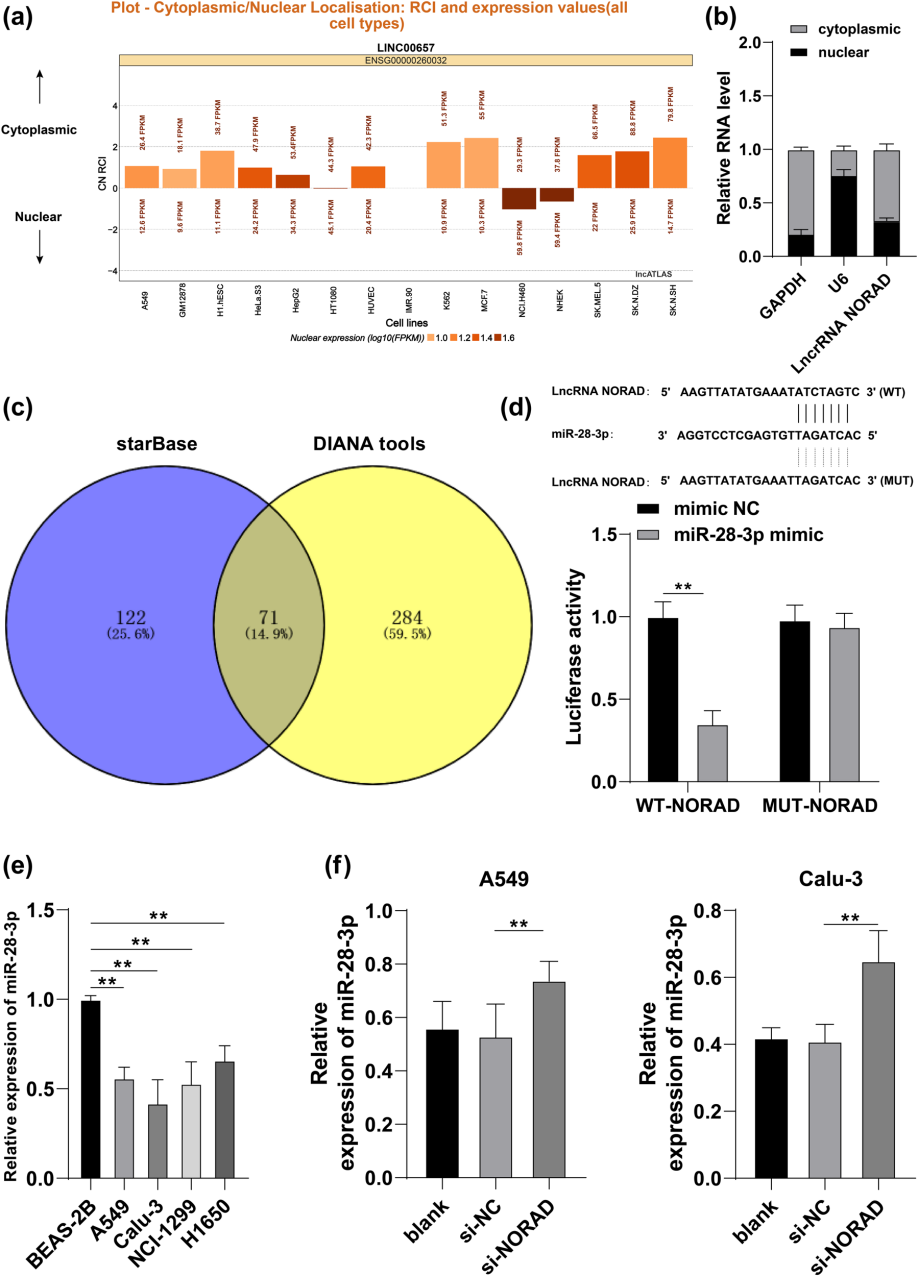
NORAD targets miR-28-3p in LC cells. (a) The subcellular localization of NORAD was predicted through the Lncatlas website (https://lncatlas.crg.eu/). (b) The subcellular localization of NORAD in LC cells was verified by fractionation of nuclear and cytoplasmic RNA assay. (c) Downstream target genes of NORAD and the intersections are exhibited by Venn diagram, with blue representing StarBase database (http://starbase.sysu.edu.cn/?tdsourcetag=s_pcqq_aiomsg) and yellow representing DIANA tools (http://carolina.imis.athena-innovation.gr/diana_tools/web/index.php?r=lncbasev2%2Findex-predicted). (d) Binding sites between NORAD and miR-28-3p and dual-luciferase reporter gene assay. (e and f) miR-28-3p expression in LC cells and BEAS-2B cells as well as cells transfected with si-NORAD was detected by RT-qPCR. The independent cell experiments were repeated three times. The results were presented as mean value ± standard deviation. Two-way ANOVA was appointed to analyze the data in panels b and d and one-way ANOVA was appointed to analyze the data in panels e and f. Tukey’s multiple comparisons test was applied for the post hoc test. ** *p* < 0.01.

To elaborately investigate the molecular mechanism of NORAD in LC cells, the subcellular localization of NORAD was predicted through the Lncatlas website (https://lncatlas.crg.eu/) and it was found that NORAD was mainly localized in the cytoplasm (Figure 2a), and fractionation of nuclear and cytoplasmic RNA assay corroborated that NORAD was mainly presented in the cytoplasm (Figure 2b). StarBase database (http://starbase.sysu.edu.cn/?tdsourcetag=s_pcqq_aiomsg) and DIANA tools (http://carolina.imis.athena-innovation.gr/diana_tools/web/index.php?r=lncbasev2%2Findex-predicted) were employed to analyze and predict the downstream miRNAs of NORAD, and then the intersection was obtained (Figure 2c), among which we focused on miR-28-3p. It has been reported that miR-28-3p is poorly expressed in a variety of cancers and affects cancer cell proliferation and migration [20,25,26]. We therefore hypothesized that NORAD modulated LC cell proliferation, invasion, and migration by regulating miR-28-3p in LC. Then, the target relation between NORAD and miR-28-3p was verified by dual-luciferase reporter gene assay (*p* < 0.05, Figure 2d). Subsequently, miR-28-3p expression in different groups was examined by RT-qPCR, and the results showed that miR-28-3p expression was reduced in A549 and Calu-3 cells compared with that in BEAS-2B cells; while it was upregulated in the si-NORAD group (*p* < 0.05, Figure 2e and f). These results supported that NORAD could bind to miR-28-3p and inhibit miR-28-3p expression.

### 3.3 miR-28-3p downregulation promotes LC cell proliferation, invasion, and migration after transfection with si-NORAD

**Figure 3:**
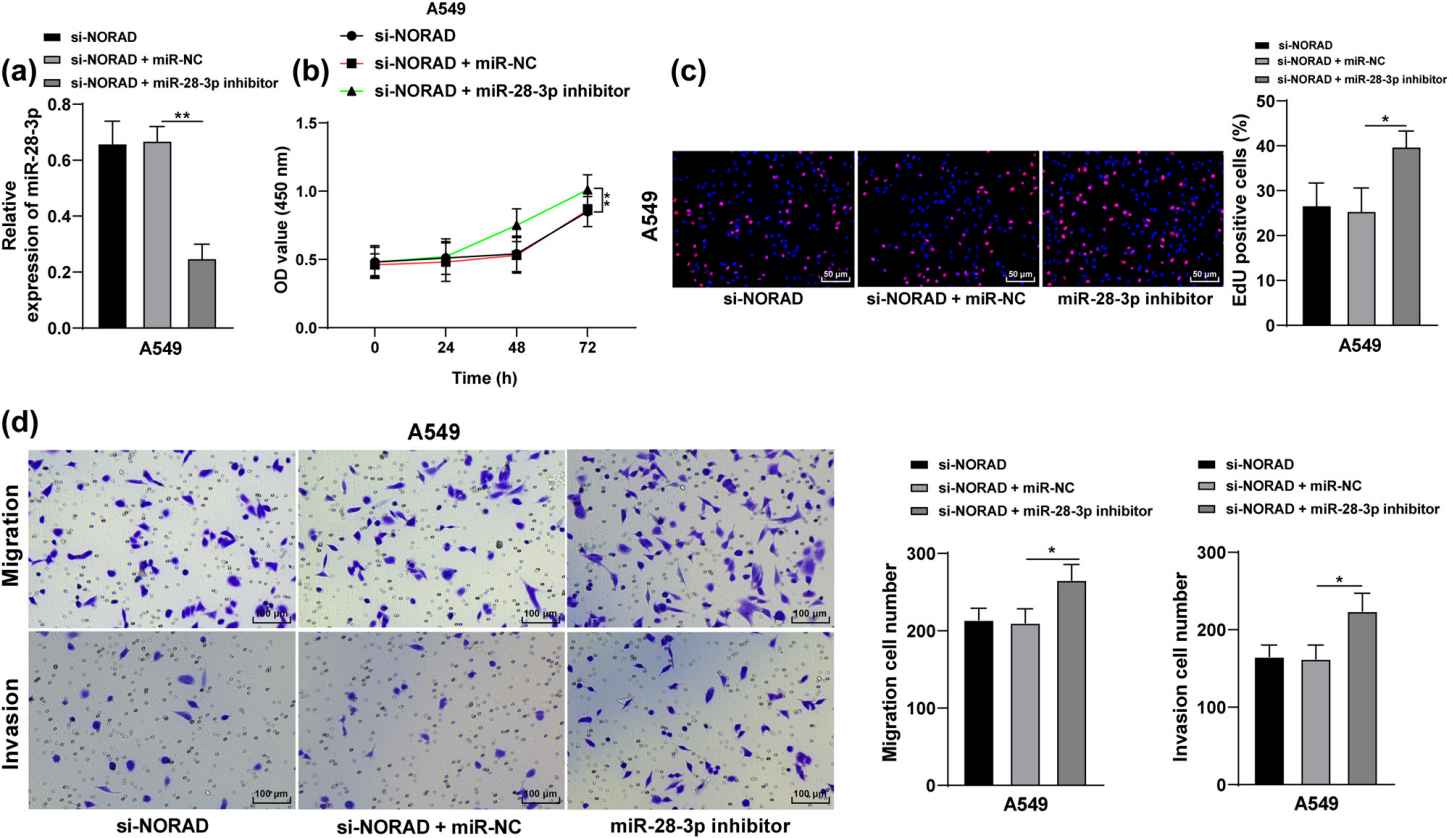
miR-28-3p downregulation promotes LC cell proliferation, invasion, and migration after transfection with si-NORAD. NORAD and miR-28-3p were simultaneously silenced in the A549 cell line. (a) miR-28-3p expression was tested by RT-qPCR. (b and c) LC cell proliferation was evaluated by the CCK-8 method (b) and EdU assay (c). (d) A549 cell invasion and migration were measured by Transwell assay. The independent cell experiments were repeated three times. The results were presented as mean value ± standard deviation. One-way ANOVA was appointed to analyze the data in panels a, c, and d, and two-way ANOVA was appointed to analyze the data in panel b. Tukey’s multiple comparisons test was applied for the post hoc test. * *p* < 0.05, ** *p* < 0.01.

In the following experiment, functional rescue experiments were carried out to verify the role of NORAD in LC cell biological behaviors. Briefly, NORAD and miR-28-3p were simultaneously silenced in the A549 cell line, and miR-28-3p expression was detected by RT-qPCR, which found that the si-NORAD + miR-NC inhibitor group had lower miR-28-3p expression than the si-NORAD + miR-NC group (*p* < 0.05, Figure 3a), and there was no statistical difference between the si-NORAD group and the si-NORAD + miR-NC group. Next experiments on cell proliferation, invasion, and migration were performed. CCK-8 method was conducted to detect cell proliferation in the 3 groups of LC cells and it was unraveled that the cell proliferation rate in the si-NORAD + miR-28-3p inhibitor group was higher than that in the si-NORAD + miR-NC group (*p* < 0.05, Figure 3b). EdU assay was used to assess cell proliferation in the 3 groups of LC cells and we noticed that cell proliferation in the si-NORAD + miR-28-3p inhibitor group was promoted compared with that in the si-NORAD + miR-NC group (*p* < 0.05, Figure 3c). And the cell invasion and migration in the 3 groups of LC cells were detected by Transwell assays, which revealed that compared with the si-NORAD + miR-NC group, the si-NORAD + miR-28-3p inhibitor group had enhanced cell invasion and migration (*p* < 0.05, Figure 3d). The experimental findings provided that miR-28-3p downregulation could promote proliferation, invasion, and migration in LC cells transfected with si-NORAD.

### 3.4 miR-28-3p targets E2F2 transcription

**Figure 4:**
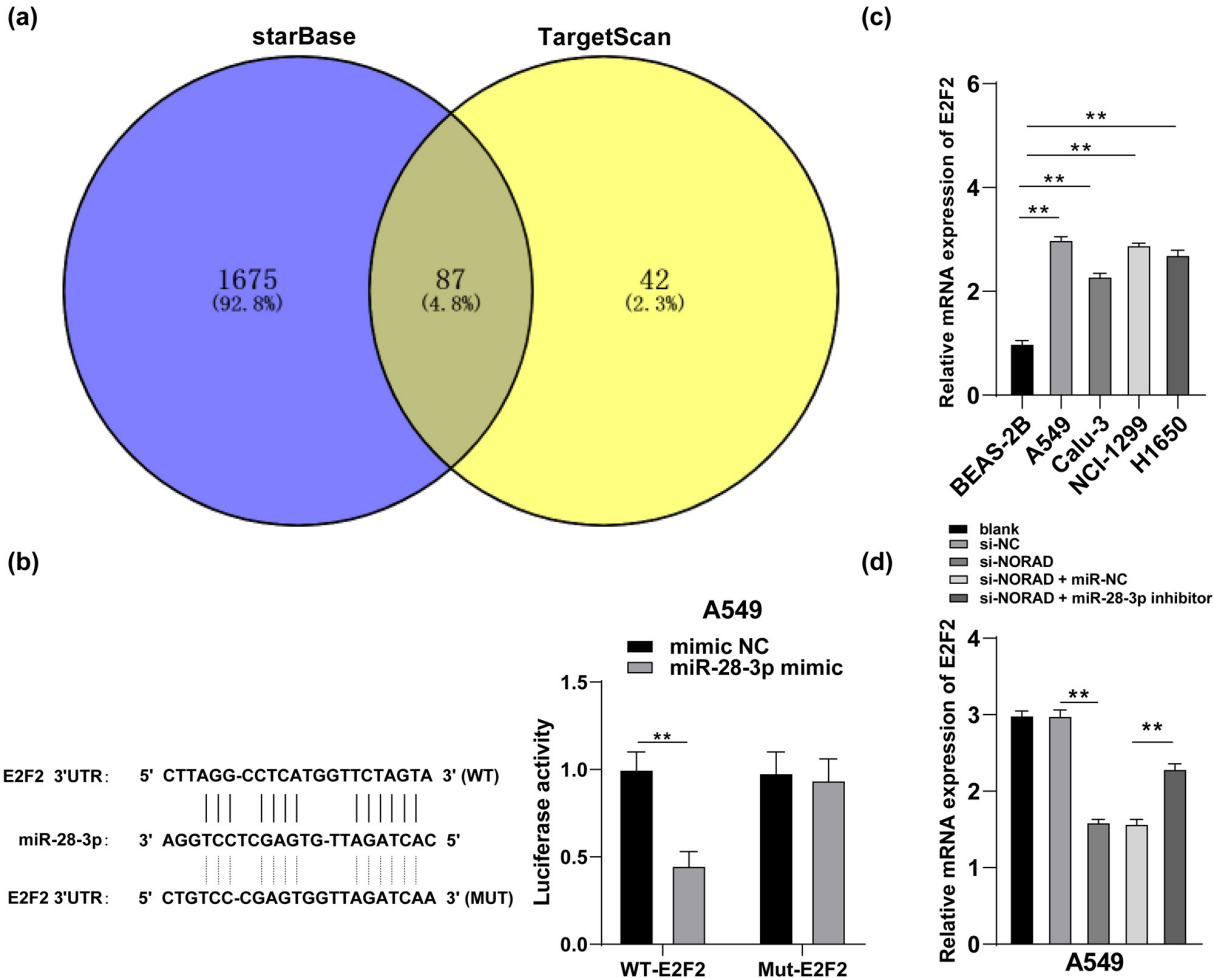
miR-28-3p targets E2F2 transcription. (a) Downstream target genes of miR-28-3p and the intersections exhibited by Venn diagram, with blue representing StarBase database (http://starbase.sysu.edu.cn/?tdsourcetag=s_pcqq_aiomsg) and yellow representing TargetScan database (http://www.targetscan.org/vert_72/). (b) Binding sites between E2F2 and miR-28-3p and dual-luciferase reporter gene assay. (c and d) E2F2 transcription level in LC cells and BEAS-2B cells, as well as A549 cells with various treatments was detected by RT-qPCR. The independent cell experiments were repeated three times. The results were presented as mean value ± standard deviation. Two-way ANOVA was appointed to analyze the data in panel b and one-way ANOVA was appointed to analyze the data in panels c and d. Tukey’s multiple comparisons test was applied for post hoc test in panels b, c, and d. ** *p* < 0.01.

To further investigate the downstream regulatory mechanisms of miR-28-3p, the downstream target genes of miR-28-3p were analyzed and predicted online through StarBase (http://starbase.sysu.edu.cn) and TargetScan databases (http://www.targetscan.org/vert_72/) and the intersection was obtained (Figure 4a), among which we focused on E2F2. E2F2 could promote LC progression [22,27]. E2F2 was confirmed as a direct target gene of miR-28-3p by dual-luciferase reporter gene assay (*p* < 0.05, Figure 4b). The transcription levels of E2F2 in different LC cell lines were then examined by RT-qPCR (*p* < 0.05, Figure 4c), which demonstrated that E2F2 expression was downregulated in response to the targeted repression of miR-28-3p (*p* < 0.05, Figure 4d). The above results suggested that miR-28-3p could target the E2F2 3′UTR region sequence and suppress E2F2 transcription.

### 3.5 E2F2 overexpression facilitates LC cell proliferation, invasion, and migration after transfection with si-NORAD

**Figure 5:**
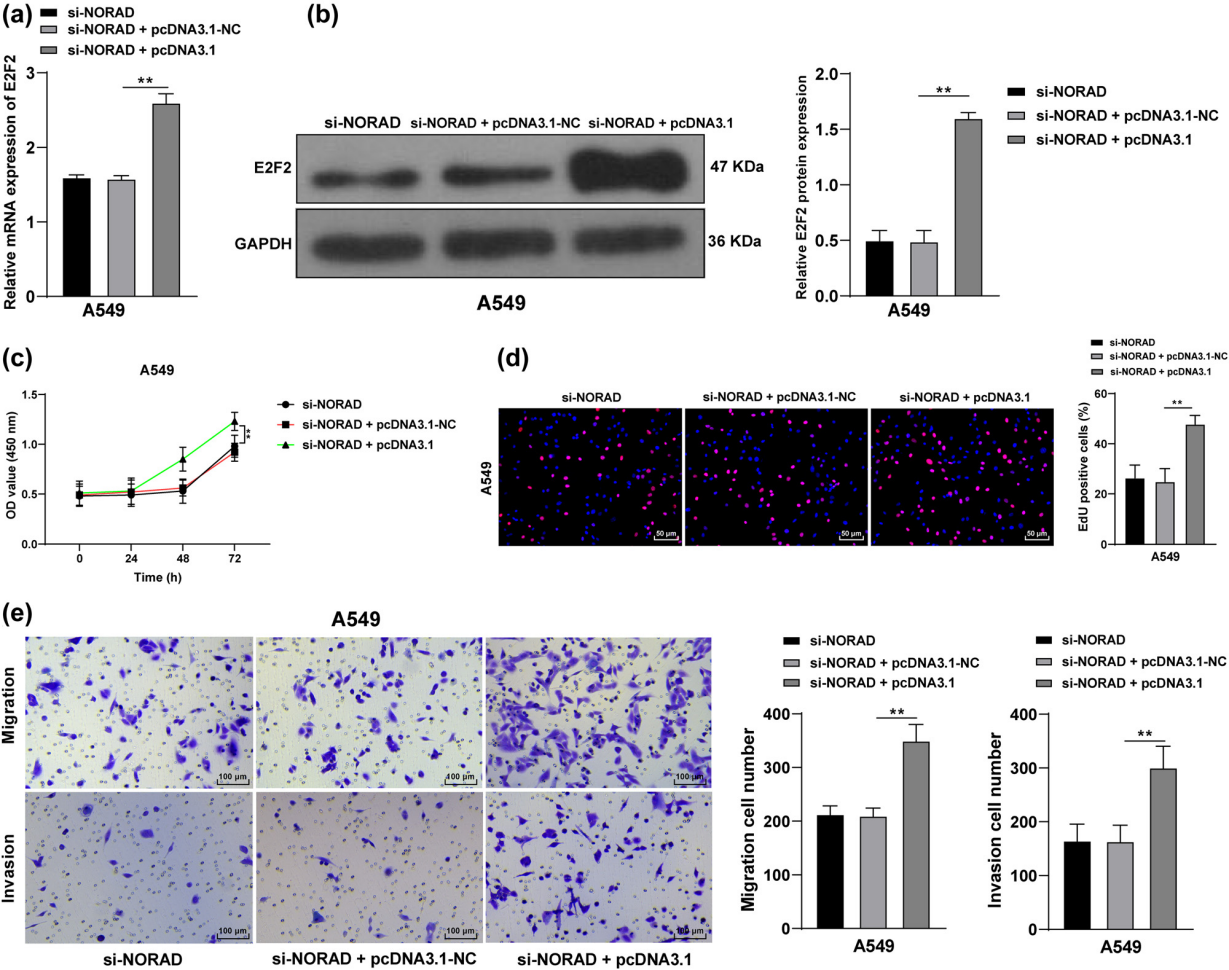
E2F2 overexpression facilitates LC cell proliferation, invasion, and migration after transfection with si-NORAD. NORAD was silenced in A549 cells in parallel with the overexpression of E2F2. (a and b) E2F2 expression in LC cells was assessed by RT-qPCR (a) and western blot analysis (b). (c and d) LC cell proliferation was examined by the CCK-8 method (c) and EdU assay (d). (e) A549 cell invasion and migration were measured by Transwell assay. The independent cell experiments were repeated three times. The results were presented as mean value ± standard deviation. One-way ANOVA was used to analyze the data in panels a, b, d, and e, and two-way ANOVA was used to analyze the data in panel c. Tukey’s multiple comparisons test was applied for post hoc test in panels b, c, and d. ** *p* < 0.01.

To further verify that NORAD mediated LC cells via the manipulation of E2F2, NORAD was silenced in A549 cells in parallel with the overexpression of E2F2. And then the transfection efficiency was verified by RT-qPCR and western blot analysis. The results found that E2F2 expression was higher in cells in the si-NORAD + pcDNA3.1-E2F2 group than that in the si-NORAD + pcDNA3.1-NC group (*p* < 0.05, Figure 5a and b), and there was no statistical difference between the si-NORAD and si-NORAD + pcDNA3.1-NC groups. CCK-8 method showed that the proliferation rate of cells in the si-NORAD + pcDNA3.1-E2F2 group was strengthened compared with that in the si-NORAD + pcDNA3.1-NC group (*p* < 0.05, Figure 5c). EdU assay unveiled that compared with the si-NORAD + pcDNA3.1-NC group, the si-NORAD + pcDNA3.1-E2F2 group showed increased cell proliferation (*p* < 0.05, Figure 5d). Transwell assays disclosed that cell invasion and migration were strengthened in the si-NORAD + pcDNA3.1-E2F2 group compared with those in the si-NORAD + pcDNA3.1-NC group (*p* < 0.05, Figure 5e). Altogether, NORAD promoted LC cell proliferation and invasion by competitively binding to miR-28-3p to encourage E2F2 transcription in LC cells.

## 4 Discussion

LC emerges as a perilous public health burden in the whole world [28]. As a kind of highly conserved and newly discovered lncRNA, NORAD is dysregulated in various cancers and is deemed as a reliable biomarker for tumor diagnosis and prognostic prediction [29]. NORAD was strongly expressed in NSCLC and encouraged NSCLC cell transformation, aggressiveness, and invasiveness [30]. Our study elucidated the role of NORAD in cancer cell proliferation, invasion, and migration of LC with the involvement of the miR-28-3p/E2F2 ceRNA network.

LncRNAs are diagnostic and prognostic target genes of NSCLC through the regulation of tumorigenesis, cell growth, and invasiveness [31]. Our first finding in this study was that NORAD was abundantly expressed in LC. Relevant research found that NORAD was strongly expressed in NSCLC and led to frustrating prognosis outcomes and severe drug resistance [32]. According to the study developed by Kawasaki, NORAD overexpression catalyzed the risky epithelial-to-mesenchymal transition of cancer cells, to stimulate lung adenocarcinoma development [33]. To further probe the effect of NORAD in LC cell biological activities, si-NORAD was injected into LC cells to reduce NORAD expression, and the results unveiled that LC cell migration, invasion, and migration were declined. Consistently, it was recently found that NORAD aggravated cell growth, mobility, and invasiveness in renal cancer [34] and gastric cancer [35]. Importantly, NORAD deficiency could sabotage cancer cell proliferation, expansion, and metastasis as well as encourage cell loss in LC [36]. However, another study by Tan and his colleagues revealed the downregulation of NORAD in LC and its association with lymph node metastasis and poor prognosis [37]. The contradiction might be associated with different cell lines and the upstream regulation of NORAD. Collectively, NORAD silencing could be conducive to the reduction in LC cell biological behaviors.

lncRNAs could participate in the ceRNA network by sponging a downstream miR, and this interaction could in turn mediate cell malignant transformation, mobility, metastasis, and invasiveness of LC [38]. To elaborately investigate the molecular mechanism of NORAD in LC cells, the subcellular localization of NORAD was predicted and NORAD was found to be mainly localized in the cytoplasm. That is to say, NORAD might function through the ceRNA interaction. It has been repeatedly reported that NORAD can intensify LC progression by sponging its downstream miRs [39,40]. MiRs are widely researched in LC malignancy since they are involved in LC tumor metastasis and aggressiveness and are potential standards for LC diagnosis, prevention, and cure [41]. Our experiments unraveled that NORAD targeted miR-28-3p. To figure out the specific role of miR-28-3p in LC, it was silenced via the injection of miR-28-3p inhibitor, after which LC cell migration, invasion, and migration were improved. miR-28-3p was insufficiently expressed in gastric cancer and predicted tumor growth, metastatic stage, cell development, and unfavorable prognostic results [26]. miR-28-5p was recognized as an anti-proliferative miR and was downregulated in hepatocellular carcinoma [42]. Moreover, miR-28-5p expression was inactivated in individuals plagued by lung squamous cell carcinoma [43]. Collectively, miR-28-3p silencing could partially neutralize the inhibitory role of si-NORAD in LC cell proliferation, invasion, and migration.

Subsequently, we noticed that miR-28-3p targeted E2F2 transcription and overexpression of E2F2 was responsible for the accelerated LC cell proliferation, invasion, and migration. E2F2 was a well-established oncogene as it was overexpressed in breast cancer and ovarian cancer and related to advanced phase, high recurrence rate, and disappointing prognosis [44,45]. E2F2 knockout was beneficial to LC cell malignant transformation, migration, and aggressiveness [46]. Essentially, as the downstream gene of a ceRNA network, E2F2 promotion induced cell viability and dissemination and reduced cell loss of LC [47]. In addition, another study about 119 LC biopsies has reported that E2F2 is preferentially expressed in adenocarcinomas subtypes vs other tumor types (squamous and others) [46]. However, as patients in this study are all from Europe and our study is specific for Chinese patients, whether there are ethnic and regional differences in E2F2 expression in LC needs more clinical data to validate. Altogether, it was concluded that NORAD affected cellular biological behaviors of LC by the ceRNA interaction of NORAD/miR-28-3p/E2F2.

In summary, our findings illustrated that NORAD impelled LC cell proliferation, invasion, and migration by competitively binding to miR-28-3p and promoting E2F2 expression. These findings hinted at a therapeutic option for LC treatment. In the future, we will further explore the underlying mechanism involved in LC and the potential therapeutic implication. Still, this is just preclinical research with some limitations. For instance, we just disclosed the molecular mechanism of the NORAD/miR-28-3p/E2F2 ceRNA interaction, but the effect of other downstream genes of NORAD or miR-28-3p is still elusive, and the role of the NORAD/miR-28-3p/E2F2 in LC cell apoptosis remains unknown. The experiment results and effective application into clinical practice need further validation and the different roles and underlying mechanisms of NORAD in different cancer types need to be further profoundly explored in the next step. And we hope our experiment could contribute some implications to the LC research.
