# Erratum to “lncRNA NORAD promotes lung cancer progression by competitively binding to miR-28-3p with E2F2”

**DOI:** 10.1515/med-2022-0626

**Published:** 2022-12-31

**Authors:** Wenjun Mao, Shengfei Wang, Ruo Chen, Yijun He, Rongguo Lu, Mingfeng Zheng

**Affiliations:** Department of Cardiothoracic Surgery, The Affiliated Wuxi People’s Hospital of Nanjing Medical University, Wuxi, 214023, Jiangsu, China; Department of Cardiothoracic Surgery, The Affiliated Wuxi People’s Hospital of Nanjing Medical University, No. 299 Qingyang Road, Wuxi, 214023, Jiangsu, China

In the published article Mao W, Wang S, Chen R, He Y, Lu R, Zheng M. lncRNA NORAD promotes lung cancer progression by competitively binding to miR-28-3p with E2F2. Open Med. (Wars) 2022 Sep 28;17(1):1538–1549. doi: 10.1515/med-2022-0538. PMID: 36245705; PMCID: PMC9520332, authors requested to replace Figure 1e.

**Figure 1:**
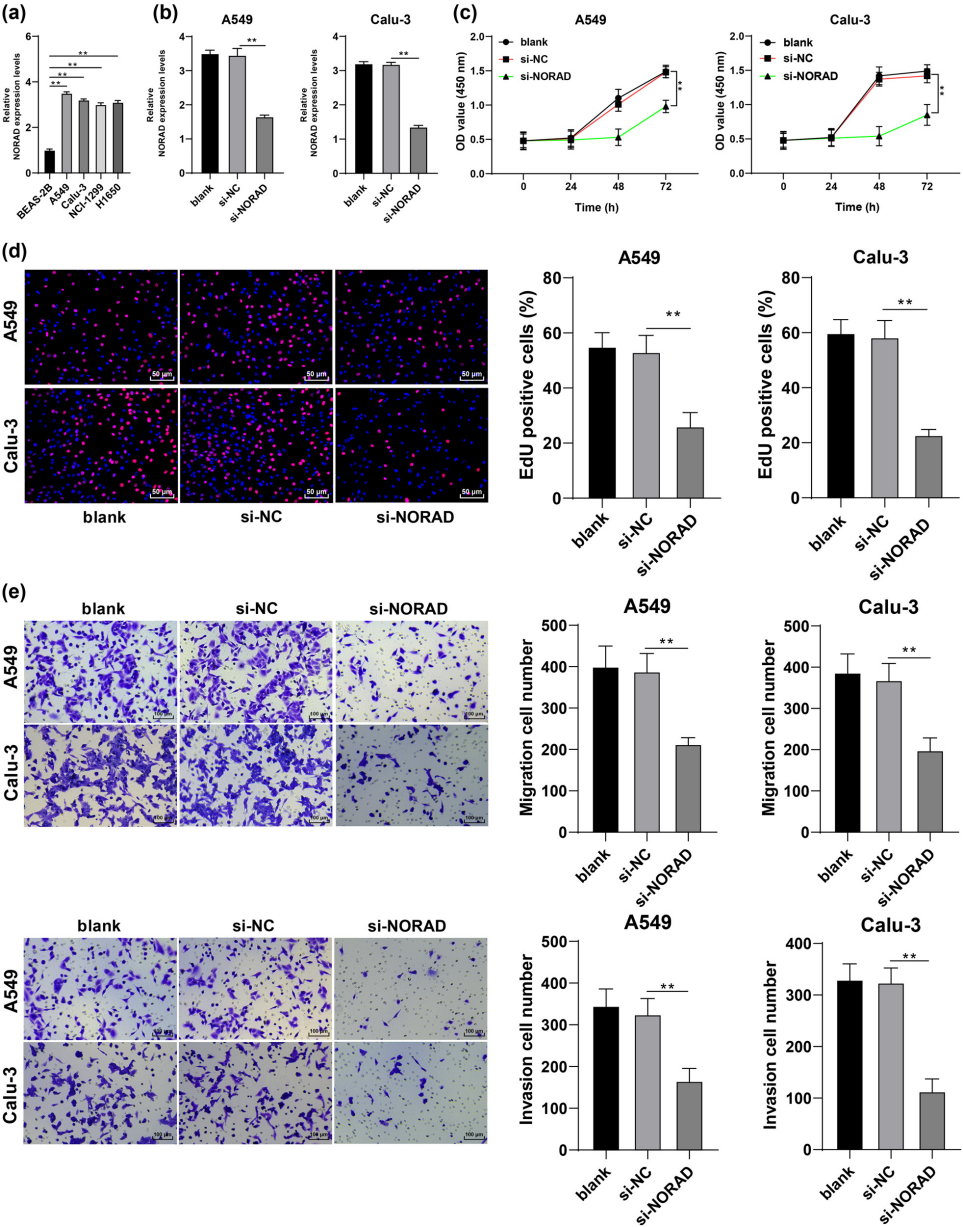
NORAD is strongly expressed in LC cell lines and NORAD knockdown in LC cells inhibits cancer cell proliferation, invasion, and migration. (a) NORAD expression in human normal lung epithelial cells (BEAS-2B) and LC cell lines (A549, Calu-3, NCI-H1299, and H1650) was assessed by RT-qPCR. si-NC or si-NORAD was transfected into A549 and Calu-3 cells. (b) NORAD expression in A549 and Calu-3 cells was assessed by RT-qPCR. (c and d) LC cell proliferation was tested by the CCK-8 method (c) and EdU assay (d). (e) LC cell invasion and migration were detected by Transwell assays. The independent cell experiments were repeated three times. The results were presented as mean value ± standard deviation. Two-way ANOVA was appointed to analyze the data in panel c and one-way ANOVA was used to analyze the data in panels a, b, and d–e. Tukey’s multiple comparisons test was applied for the post hoc test. **p < 0.01.

After publication, the authors found out that due to their negligence, the image of A549 cell invasion after the treatment of si-NORAD + pcDNA3.1 was wrongly used as the image of A549 cell invasion after the treatment of si-NC of Figure 1e. Now, the authors would like to publish the correct Figure 1e.

The correct Figure 1 is presented as follows:

